# Natural product nanozymes of herbal extract galangin in managing hepatocellular carcinoma

**DOI:** 10.3389/fchem.2024.1426634

**Published:** 2024-06-10

**Authors:** Erhao Wang, Yuxia Wu, Yan Wang, Jiao Li, Xiuzhen Liang, Zhongtao Wang, Xiaofei Liu, Faming Feng, JianCang Mao, Yingqi Zhu, Le Li

**Affiliations:** ^1^ Hainan Women and Children’s Medical Center, Haikou, China; ^2^ Hainan General Hospital/Hainan Affiliated Hospital of Hainan Medical University, Haikou, China; ^3^ NHC (National Health Commission of the People’s Republic of China) Key Laboratory of Tropical Disease Control, School of Tropical Medicine, Hainan Medical University, Haikou, Hainan, China

**Keywords:** galangin, nanozyme, reactive oxygen species, apoptosis, fenton reaction

## Abstract

Numerous local herbal extract species have been investigated as potential medicinal ingredients due to their promising anti-cancer properties. However, the primary constraint of the class of plant flavonoids lies in their low solubility and limited membrane permeability, leading to chemical instability and restricted bioavailability that impede biomedical applications. In this study, we have developed an ideal nanozyme-Galazyme, comprising galangin-loaded copper Nanozyme coated by DSPE-PEG, which amplifies oxidative stress to induce apoptosis via the regulation of reactive oxygen species (ROS) generation and mitogen-activated protein kinase (MAPK) activation. Galazyme exhibited significant peroxidase mimetic activity, demonstrating its potential to generate ROS and elevate oxidative stress. Upon uptake by HepG-2 cells, Galazyme efficiently converts excess hydrogen peroxide (H2O2) into highly reactive •OH radicals and upregulates MAPK expression, leading to the activation of Bax and Caspase 3, thereby promoting irreversible tumor cell apoptosis. Both *in vitro* and *in vivo* results demonstrate that Galazyme inhibits tumor cell growth and induces apoptosis by generating ample ROS and activating the MAPK pathway. Our study offers novel evidence supporting the enhancement of Galazyme-induced apoptosis through the upregulation of Bax and Caspase 3, along with the elucidation of the interaction between MAPK and apoptosis.

## 1 Introduction

Flavonoids, as one of the most common herbal metabolites, have garnered significant attention in the treatment of various diseases (Ren et al., 2003; Kandaswami et al., 2005; Zhu et al., 2024a; Chen et al., 2024). Flavonoids and derivatives have been demonstrated to possess a range of biological properties, including antitumor, antioxidant, antibacterial, and anti-inflammatory activities (Cushnie and Lamb, 2005; Farhadi et al., 2019; Zhu et al., 2024b). Interestingly, numerous preclinical studies have shown that flavonoids can modulate various forms of regulated cell death (RCD) (Wu et al., 2019; Wu et al., 2022). Regulated cell deaths (RCDs), such as pyroptosis, apoptosis, necroptosis, and ferroptosis, play crucial roles in different stages of disease progression and are closely linked to treatment strategies (Tang et al., 2019; Niu et al., 2023). Galangin (3,5,7-trihydroxyflavone) is regarded as the bioactive constituent of galangal, a perennial plant native of Indonesia but cultivated in many parts of Asia. The anticancer effects of galangin are mostly due to its abilities to inhibit cell cycle progression, inhibiting mitogen-activated protein kinase (MAPK) (Liu et al., 2015), protein kinase B (Akt) (Li et al., 2018), or mammalian target of rapamycin (mTOR) activity thereby inducing apoptotic cell death by activating caspase-9/-8/-3 and inhibiting tumor invasion and metastasis by reducing the upregulation of matrix metalloproteinase-2/-9 (Kong et al., 2019; Wu et al., 2024). These molecular pathways regulated by galangin are involved in suppressing different malignancies, such as lung cancer, hepatic cancer, ovarian cancer, breast cancer, gastric cancer, colorectal cancer, retinoblastoma, and osteosarcoma (Liang et al., 2021). Based on the effective tumor intervention, the rational development of new drug formulations related to galangin is of great significance in overcoming the challenges of RCD-related tumor treatment.

Nanozyme, nanoparticle endowed with inherent enzyme-mimic activities, has motivated the rapid advancement for novel tumor therapy strategy (Huang Y. et al., 2019; Zhu et al., 2021a; Tang et al., 2021; Zhu D. et al., 2022; Zhu et al., 2023). Nanozyme emerge as a promising candidate for regulating cellular bioprocesses due to their satisfactory enzymatic activities, excellent stability, cost-effectiveness, and superior membrane permeability compared to natural enzymes (Zhu et al., 2021b; Xu et al., 2022; Wang et al., 2023). A great deal of nanozymes have been reported to display impressive catalytic performances for cancer therapy (Jiang et al., 2019; Zhu Y. et al., 2021). Significantly, nanozymes with peroxidase-catalytic activity (POD-like activity), such as copper nanozymes (Zhu Y. et al., 2022; Niu et al., 2023), single atom nanozymes, manganese nanozymes, and iron nanozymes (Huang L. et al., 2019; Cheng et al., 2021), have been employed to generate sufficient reactive oxygen species (ROS) to destroy tumor cells. Chen et al. present a chemodynamic nanomedicine, comprising oleanolic acid-loaded iron single atom nanozyme coated by red cell membrane, which enhances lipid peroxidation to boost anticancer efficacy via the regulation of cell membrane unsaturation. Therefore, nanozyme can serve as a potential therapeutic agent for conquering clinical cancer treatment challenges.

**SCHEME 1:**
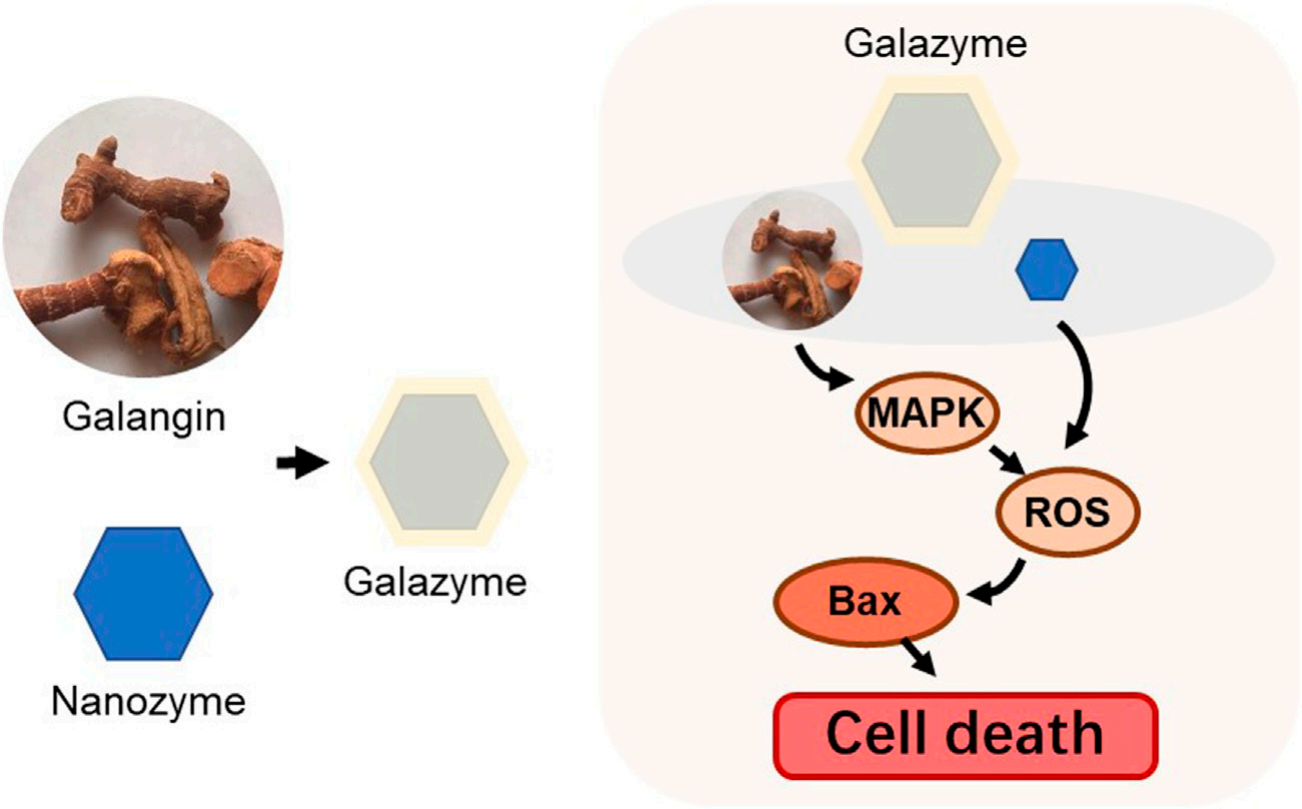
Schematic illustration of the fabrication of the Galazyme.

In this study, we developed an impressive nanozyme-Galazyme (Scheme 1), comprising galangin-loaded copper Nanozyme coated by DSPE-PEG, which strengthens oxidative stress to induce apoptosis by regulating ROS initiation and MAPK activation. Galazyme exhibited significant peroxidase mimetic activity, demonstrating its potential to generate ROS and elevate oxidative stress. Upon uptake by cancer cells, Galazyme not only catalytically converted overproduced hydrogen peroxide (H_2_O_2_) into highly active •OH but also activated the MAPK pathway, leading to the upregulation of Bax and Caspase 3, thereby promoting irreversible apoptosis. Both *in vitro* and *in vivo* results demonstrated that Galazyme suppressed tumor cell growth and induced tumor apoptosis through initiating ROS and activating MAPK pathway. Our work offers a new proof-of-concept to construct natural product Nanozymes which enhance apoptosis via upregulating Bax and Caspase three while establishing MAPK and apoptosis interaction.

## 2 Materials and methods

### 2.1 Materials

Galangin was purchased from MedChemExpress (MCE, China). Dimethylimidazole, copper acetylacetonate, and zinc nitrate hexahydrate were bought from Sigma. Hoechst, Annexin V-FITC, propidium iodide (PI), 2′,7′-dichlorofluorescin diacetate (DCFH-DA) and cell count kit-8 (CCK-8), were bought from Beyotime (Shanghai, China). Paraformaldehyde (PFA), Tween 20, Triton X-100 were purchased from Sinopharm Chemical Reagents (Shanghai, China). *Mycoplasma* Removal Agent (cat #60703) were purchased from Yeasen, Shanghai, China. Deionized (DI) water was obtained from a Milli-Q water purification system.

### 2.2 Synthesis of galazyme

Solution A consisted of 2.36 g of dimethylimidazole and 200 mg of copper acetylacetonate dissolved in 80 mL of methyl alcohol, while solution B comprised 1.07 g of zinc nitrate hexahydrate dissolved in 80 mL of methyl alcohol. Solution B was then slowly added to solution A under magnetic stirring and mixed for 6 h. The resulting product was collected by centrifugation and washed with methyl alcohol three times. Finally, the product underwent vacuum drying at 60°C. Subsequently, the product was subjected to pyrolysis at 600°C for 2 h to yield Nanozyme.

The 10 mg Nanozyme was dissolved in 20 mL of 20 mL DD H_2_O. Separately, 2 mg of galangin was dissolved in 1 mL of dimethyl sulfoxide (DMSO). The galangin-containing DMSO solution was then added to the Nanozyme solution. Then, the Galazyme was collected by centrifugation and washed with PBS for three times.

### 2.3 Drug loading and entrapment efficiency

The galangin were loaded into Nanozyme with a simple impregnation method under magnetic stirring. Then, it was subjected to ultracentrifugation for 2 h at 6,000 r. The amount of drug load and entrapment efficiency (EE) within EVs were quantified using a spectrophotometer (UV-2600, SHIMADZU, Kyoto, Japan). The EE was calculated according to the following Equations:

$$\%\text{ EE}=\left[\left(\text{Drug added }‐\text{ unloaded Drug}\right)/\text{ Drug added}\right]\times 100$$

### 2.4 Cellar uptake

HepG2 cells were seeded in confocal dishes for 12 h. After 4 h of incubation with Galazyme, tumor cells were collected and the content was measured by high-performance liquid chromatography (HPLC).

### 2.5 Proliferation assay

Cell viability was measured by the CCK-8 assay. HepG2 cells were planted for 24 h. Then, the cells were incubated with various concentrations of Galazyme. After treatment for 24 h, the medium was replaced with fresh medium containing 10 μL CCK-8 and quantified by the absorbance at 450 nm using a microplate reader.

### 2.6 Reactive oxygen species (ROS) generation

HepG2 cells were seeded in confocal dish. The cells were treated with different formulations for 4 h. Then, the cells were co-stained with DCFH-DA (10 μM) and Hoechst (10 μM). The fluorescence imaging of cells was imaged by confocal microscopy.

### 2.7 *In vivo* anti-tumor assay

Animal experiments were performed according to the protocol approved by The Ethical Committee of Hainan Medical University. HepG2 tumor-bearing mice were randomly divided into four groups (5 mice per group) and intravenously administrated with 5 mg/kg Galazyme for 3 times every 3 days. After 14 days of treatment, the mice were euthanatized for histological examination.

### 2.8 Statistical analysis

Data represent the mean ± s.d. From indicated independent replicates. Statistical analysis was conducted using GraphPad Prism. For comparisons between two groups, means were compared using the unpaired two-tailed Student’s t-test. A value of *p* < 0.05 was considered statistically significant.

## 3 Results and discussion

### 3.1 Synthesis and characterization of galazyme

**FIGURE 1:**
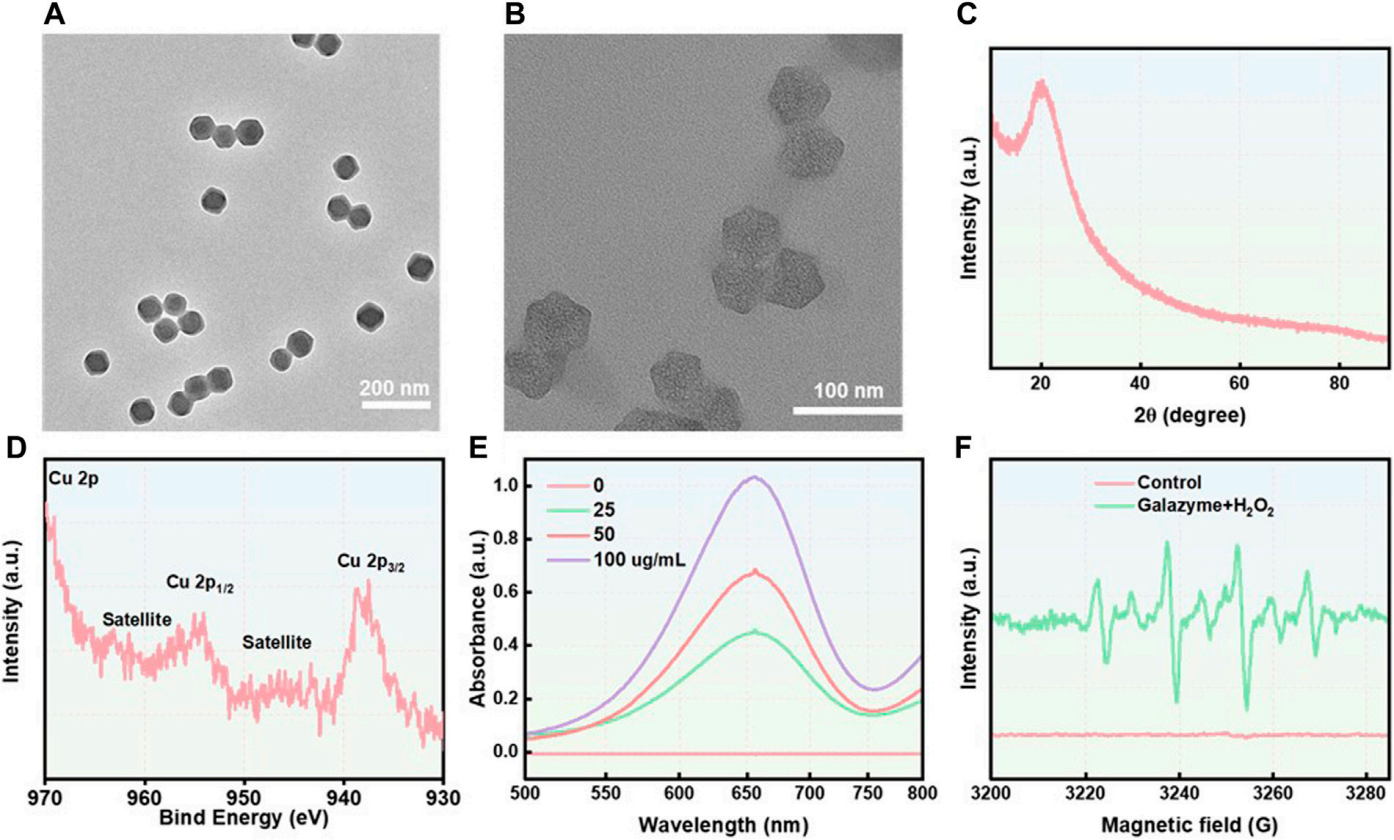
**(A)** The TEM image of ZIF-8. Scale ber: 200 nm. **(B)** The TEM image of Nanozyme. Scale ber: 100 nm. **(C)** The XRD spectrum of Galazyme. **(D)** The XPS spectrum of Galazyme. **(E)** The UV-vis spectra of TMB treated with Galazyme and H_2_O_2_ at different concentrations. **(F)** The ESR spectra of •OH trapped using DMPO.

In this study, ZIF-8 nanoparticles, with a particle size of approximately 40 nm, were fabricated using a Host-Guest Template and observed via transmission electron microscopy (TEM) (Figure 1A). The TEM image revealed that there are numerous pores on the surface of Nanozyme, attributing to the efficient loading of the galangin (Figure 1B). The zeta-potential of the nanoparticles was measured as ZIF-8: 21.3 mV, Galazyme: 20.5 mV. The long-term measurement of the stability was studied by dynamic light scattering (DLS). The DLS result showed that the diameter of Galazyme remained stable for 48 h, which indicated the stability of Galazyme in biological environments (Supplementary Figure S1). No apparent crystal peak was noticed in the X-ray powder diffraction (XRD) spectrum, implying the poor crystallinity of Cu element in Galazyme (Figure 1C). And the X-ray photoelectron spectroscopy (XPS) was selected to measure the Cu valences in Galazyme. As shown in Figure 1D, Cu 2p XPS spectrum showed that two characteristic Cu peaks at Cu^2+^ (937.5 eV) and Cu^+^ 954.1 eV, implying the remarkable Fenton reactivity of Galazyme. In addition, the Fenton activity of Galazyme was assessed using 3,3′,5,5′-tetramethylbenzidine (TMB) as a chromogenic substrate. As shown in Figure 1E, Galazyme converted TMB into oxidized TMB (ox-TMB) with absorbance at 652 nm and acidic pH in the presence of hydrogen peroxide (H_2_O_2_), implying the pH-dependent catalytic activity of Galazyme. The peroxidase (POD) activity of Galazyme in different pH were studied by UV-vis. The results demonstrated that Galazyme can convert TMB into oxidized TMB (ox-TMB) under acidic pH while no obvious chromogenic reaction was observed at neutral pH 7.4, indicating that Galazyme exhibited a pH-dependent Fenton reaction to produce plentiful ROS (Supplementary Figure S2). Furthermore, the Fenton activity of Galazyme exhibited a concentration-dependent manner. Moreover, 5,5-Dimethyl-1-pyrroline N-oxide (DMPO), a spin trapper reacting with the intermediate •OH, was employed as the •OH product indicator. Electron spin resonance (ESR) spectrum demonstrated a characteristic quartet signal (1:2:2:1) of DMPO/•OH adduct when Galazyme was treated with H_2_O_2_ at acidic pH (Figure 1F). These finding confirmed the catalytic activity of Galazyme and its enhanced potential to initiate a ROS storm.

### 3.2 *In Vitro* cytotoxicity of galazyme

**FIGURE 2:**
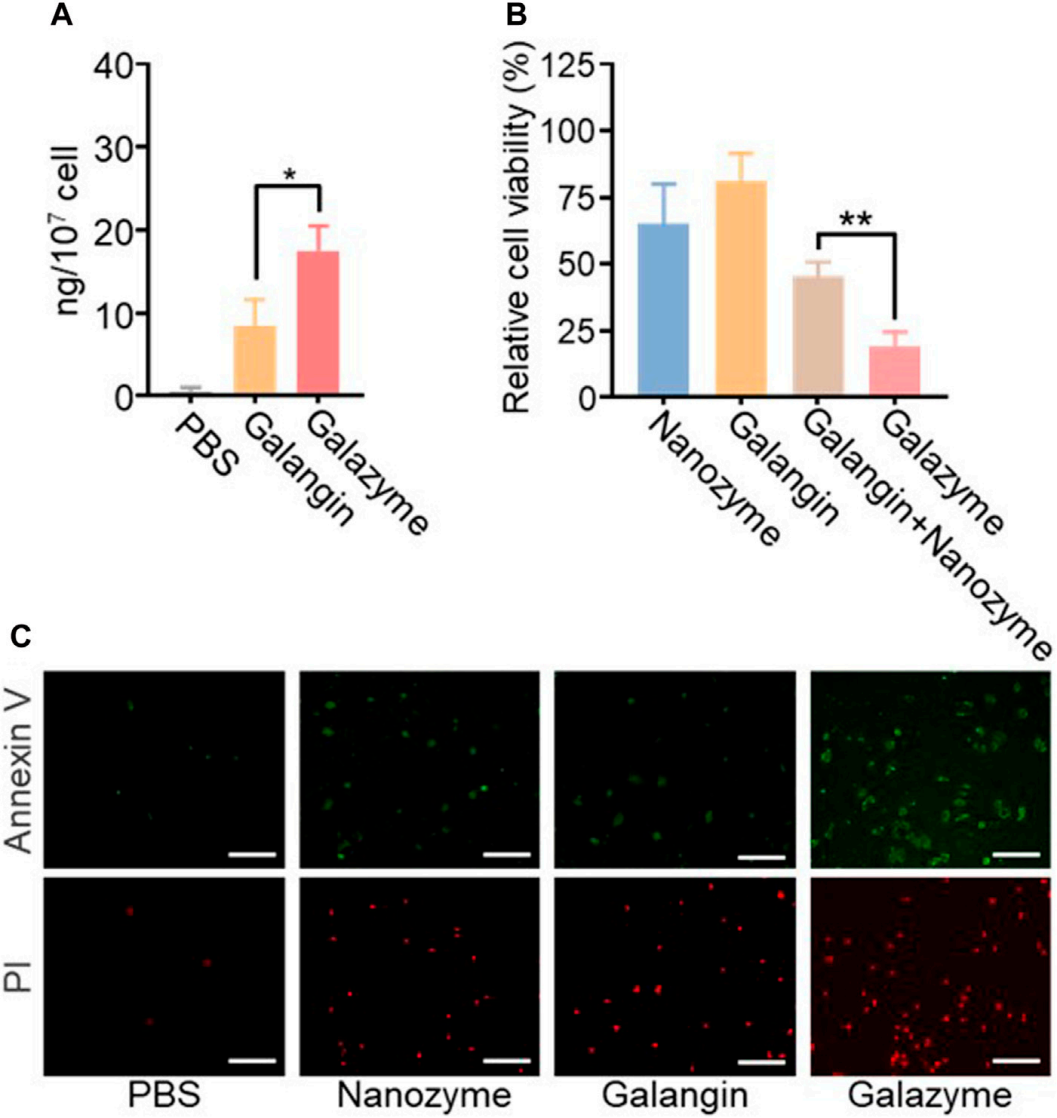
**(A)** Intracellular content analysis of the treatment of Galangin and Galazyme. **(B)** Cell viability analysis of cells after the indicated treatments. The drug concentration was based on 10 μM galangin. Data are expressed as mean ± SEM of three independent experiments. **(C)** Cell apoptosis analysis of cells after the treatment of PBS, Nanoenzyme, Galangin, and Galazyme. Scale bar: 100 μm. (*p < 0.05, **p < 0.01).

The *in vitro* anti-proliferation effect was evaluated using various assays based on the impressive excellent catalytic activity of Galazyme and the galangin-induced chemotherapy toxicity. The cellular uptake of Galazyme and galangin were quantified by HPLC. Figure 2A demonstrated that Galazyme showed higher internalization efficiency compared to free galangin, ascribing to the passive uptake of nanoparticles by tumor cells. The cell counting kit-8 (CCK-8) assay was conducted to quantitatively measure the *in vitro* cellular cytotoxicity of Galazyme on HepG2 cells. As shown in Figure 2B and Supplementary Figure S3, free nanozyme or galangin partially inhibited tumor cell growth, whereas Galazyme markedly suppressed it, validating its remarkable catalytic activity and enhanced chemotherapy toxicity. In addition, the bright fields images of tumor cells were imaged to assess the cytotoxicity of Galazyme. As anticipated, Galazyme can effectively induce tumor cell death (Supplementary Figure S4). Furthermore, the live/dead assay was employed to evaluate the tumor cell-killing effect of Galazyme using the Annexin-V/propidium iodide (PI). Galazyme demonstrated remarkably brighter fluorescence (indicating apoptosis) than the Nanozyme or galangin alone group (Figure 2C; Supplementary Figure S5). The cytometry analysis further proved the tumor cell killing effect (Supplementary Figure S6). These finding confirmed that Galazyme inhibited tumor cell growth via synergetic catalytic therapy and chemotherapy toxicity.

### 3.3 Therapeutic mechanisms of galazyme-induced cell death

**FIGURE 3:**
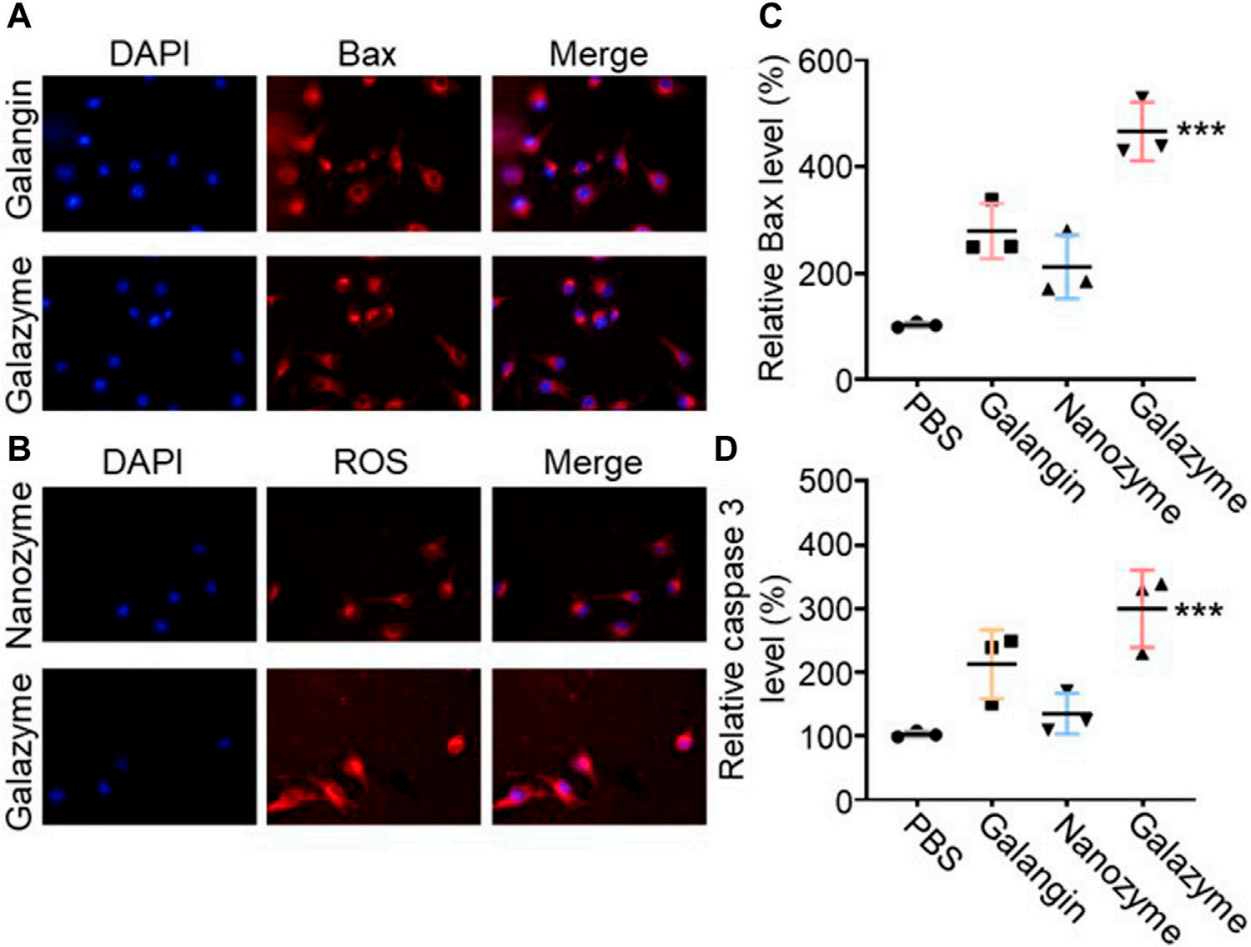
**(A)** The phosphorylated MAPK levels in tumor cells after treatment with different formulations Nucleus was stained with DAPI. **(B)** The ROS levels in tumor cells after treatment with different formulations. Nucleus was stained with DAPI. **(C)** Bax expression in tumor cells treated with different formulations. **(D)** Cleaved caspase three expression in tumor cells treated with different formulations. (***p < 0.001).

The releated tumor cell death pathway was investigated to assess the potential mechanism of Galazyme-induced therapeutic efficacy. As previous reported, galangin can effective upregulate the MAPK expression to induce tumor cell apoptosis. The immunofluorescence staining was employed to measure the phosphorylated MAPK levels in tumor cells after treatment with different formulations. As shown in Figure 3A, both galangin and Galazyme effective elevated the expression in tumor cells. Then the excessive cellular ROS level was assessed using a fluorescence probe (2′,7′-dichlorofluorescin diacetate, DCFH-DA). It can be seen in Figure 3B that Galazyme displayed brighter red fluorescence intensity compared to free galangin, attributing to the excellent catalytic activity. Moreover, the qRCR was conducted to measure the Bax level and Caspase 3 level in tumor cells after treated with different formulations. As expected, Galazyme greatly elevated the expression of Bax and Caspase-3, ascribing the satisfactory •OH generation and MAPK activation (Figures 3C, D). These results indicated that Galazyme convert H_2_O_2_ into highly toxic •OH and activated MAPK pathway, inducing tumor cell apoptosis.

### 3.4 *In vivo* antitumor effect of galazyme

**FIGURE 4:**
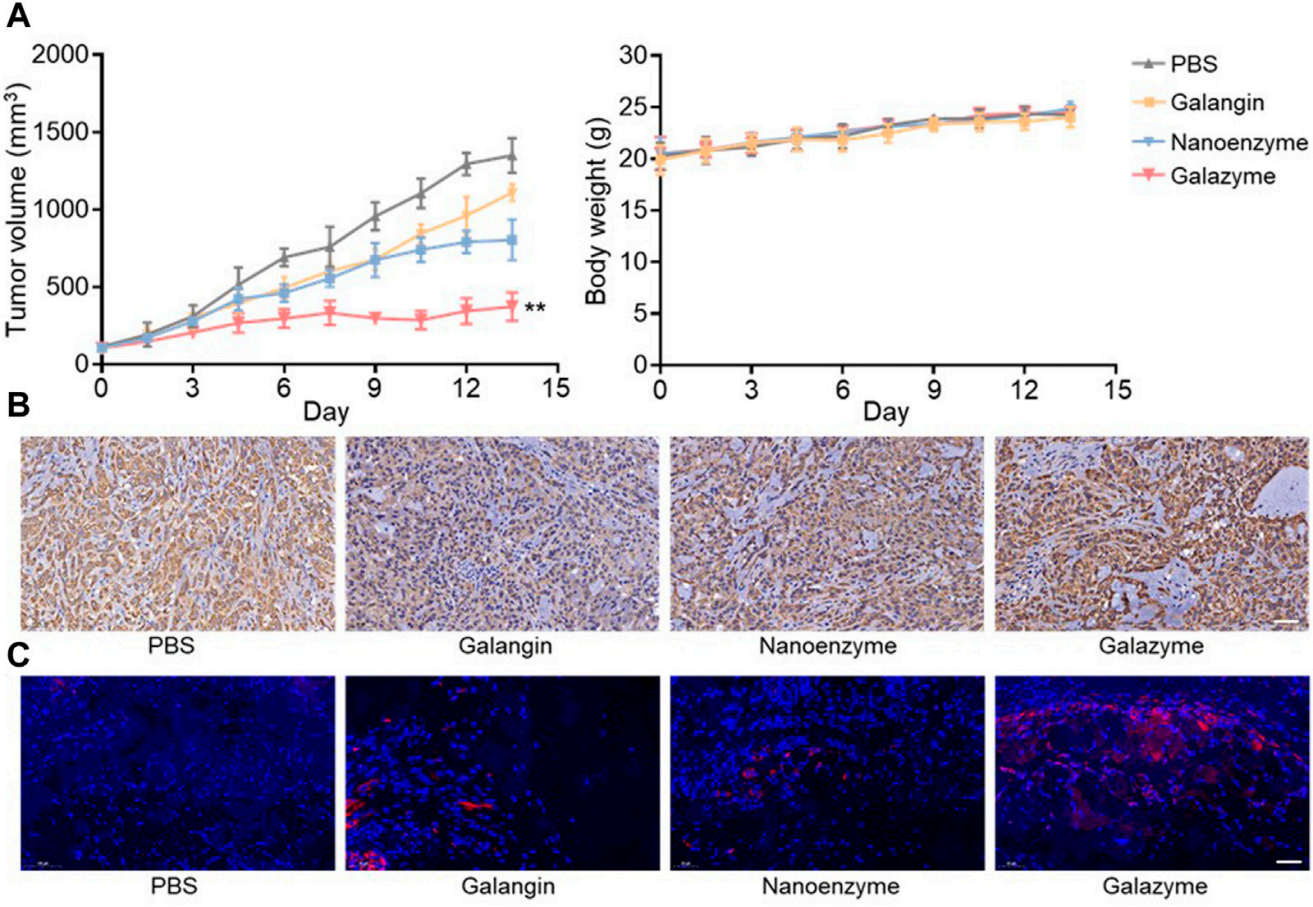
**(A)** Tumor volume and body weight change of each treatment group. **(B)** Images of immunochemical staining TUNEL assays of tumor tissues after different treatments. Scale bar: 50 μm. **(C)** Images of immunofluorescence staining of the MAPK. Nucleus was stained with DAPI. Scale bar: 50 μm. (**p < 0.01).

The Ethical Committee of Hainan Medical University approved the protocol for the animal experiments. The synergistic therapeutic efficacy triggered by Galazyme was studied in tumor-bearing nude mice post-intravenous injection. The tumor-bearing nude mice were randomly divided into four groups: PBS, Galangin, nanozyme, and Galazyme groups. As can be seen in Figure 4A, Nanozyme partially suppressed tumor growth, ascribed to the impressive Fenton activity. In comparison, Galazyme demonstrated a significant inhibition of tumor growth, attrubuted to the synergistic chemodynamic and apoptosis involving ROS generation (Supplementary Figure S7). Importantly, no significant variation in body weight was observed among the different groups, suggesting the favorable biosafety profile of Galazyme. Moreover, the antitumor efficacy of Galazyme was confirmed through immunochemical TUNEL and immunofluorescence staining of tumor sections. Figure 4B showed that Galazyme induced the highest level of cell death and activated MAPK expression compared to free galangin and Nanozyme. And Figure 4C revealed that Galazyme effectively induced the MAPK activation in tumor cells. Collectively, these results supported the enhanced antitumor efficacy of Galazyme via activating MAPK pathways and generating plentiful ROS.

## 4 Conclusion

In conclusion, we developed an impressive nanozyme-Galazyme with MAPK activation and ROS production capabilities. Upon uptake of cancer cells, Galazyme not only catalytically converted overproduced H_2_O_2_ into highly active •OH but also activated MAPK pathway, causing the upregulation of Bax2 and Caspase 3, which in return promoted irreversible apoptosis. Both *in vitro* and *in vivo* results revealed that Galazyme suppressed tumor cell growth and triggered tumor apoptosis through initiating ROS and activating MAPK pathway. Our work highlights the potential of Galazyme as a promising apoptosis nanomedicine to deliver effective tumor therapy.

## Data Availability

The original contributions presented in the study are included in the article/Supplementary Material, further inquiries can be directed to the corresponding author.
