# Supplementary material for: Natural product nanozymes of herbal extract galangin in managing hepatocellular carcinoma
Source: Front Chem. 2024 Jun 10;12:1426634. doi: 10.3389/fchem.2024.1426634 (PMC11194749; doi:10.3389/fchem.2024.1426634)
Supplement: Supplementary file 1 [file DataSheet1.docx]

Supplementary Material

Natural Product Nanozymes of Herbal Extract Galangin in Managing Hepatocellular Carcinoma

Wang Erhao^1^, Wu Yuxia^2^, Wang Yan^1^, Li Jiao^1^, Liang Xiuzhen^1^, Wang Zhongtao^1^, Liu Xiaofei^1^, Feng Faming^1^, JianCang Mao,^3^ Yingqi Zhu,^3^ Le Li^3*^

^1^ Hainan Women and Children’s Medical Center, Haikou, China

^2^ Hainan General Hospital/Hainan Affiliated Hospital of Hainan Medical University, Haikou, 570311, China

^3^ NHC（National Health Commission of the People's Republic of China）Key Laboratory of Tropical Disease Control, School of Tropical Medicine, Hainan Medical University, Haikou, Hainan, 571199, China

# Supplementary Figures

**Supplementary Figure 1.** The stability of the Galazyme in DMEM.

**Supplementary Figure 2.** The POD activity of Galazyme in different pH.


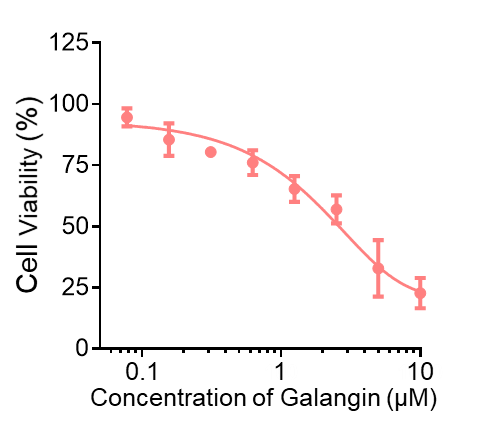


**Supplementary Figure 3.** Cell viability analysis of cells after the treatment with Galanzyme. The drug concentration was based on galangin.


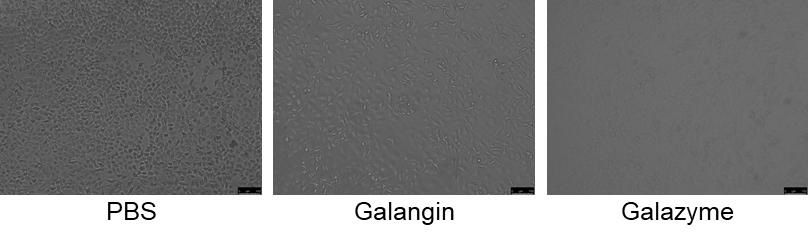


**Supplementary Figure 4.** Images of HepG2 cell after treated with different drugs.


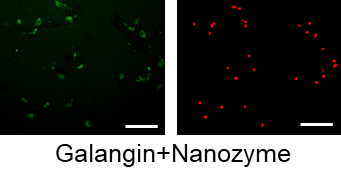


**Supplementary Figure 5.** Cell apoptosis analysis of cells after the treatment of the drug mixture. Scale bar: 100 μm.


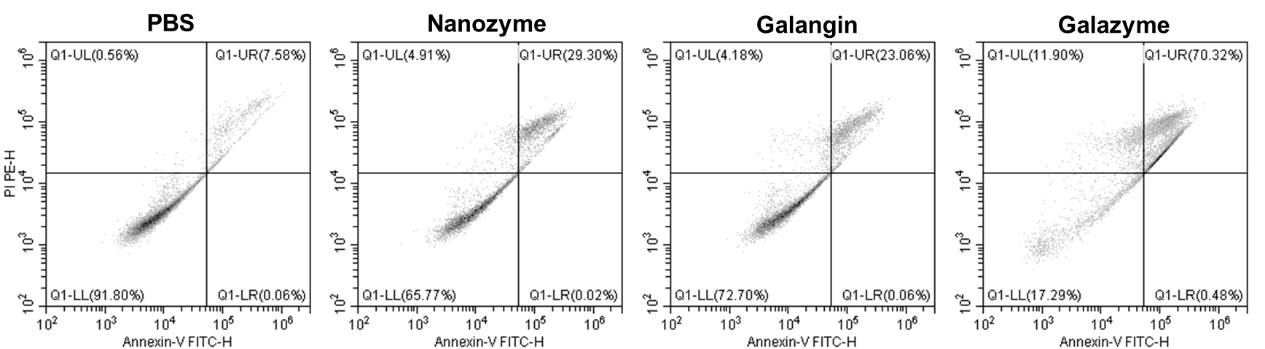


**Supplementary Figure 6.** Cell apoptosis analysis by flow cytometry.


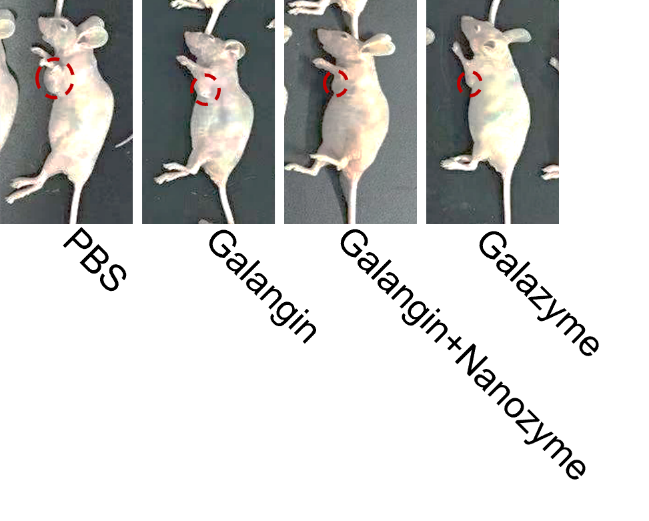


**Supplementary Figure 7.** The tumor weight analysis and photos of the tumor after different treatments.
